# Pulsed field ablation as first-line therapy for the treatment of atrial fibrillation: a FARADISE sub-analysis

**DOI:** 10.1093/europace/euag275

**Published:** 2026-10-14

**Authors:** Ignacio García-Bolao, Nándor Szegedi, Johan Vijgen, Gábor Széplaki, Antonio Dello Russo, Michael Efremidis, Stephan Willems, Haris Haqqani, Estelle Gandjbakhch, Martin Fiala, Georgios Andrikopoulos, Armin Luik, Pascal Defaye, Alexandre Ouss, Patrick Lugenbiel, Pawel Balsam, Alan Bulava, Michael Gramlich, Lars Eckardt, Azlan Hussin, Claire A Martin, Mattias Duytschaever, Joaquín Osca, Alexis Elfstrum, Nele Cielen, Madeline Johnson, Angelo Auricchio, Lucas V A Boersma

**Affiliations:** Clinica Universidad de Navarra, Avenida Pío XII, 36, Pamplona 31008, Spain; Heart and Vascular Center, Semmelweis University, 68 Városmajor Street, Budapest H-1122, Hungary; Jessa Hospital, Hasselt, Belgium; Mater Private Hospital and Royal College of Surgeons in Ireland, Dublin, Ireland; Biomedical Science and Public Health Department, Marche University Hospital, Ancona, Italy; Onassis Cardiac Surgery Center, Kallithea, Greece; Asklepios Clinic St. Georg, Hamburg, Germany; Prince Charles Hospital, Chermside, Australia; Sorbonne Université, APHP, La Pitié Salpetrière University Hospital, Paris, France; Neuron Medical, Brno, Czechia; Henry Dunant Hospital Center, Athens, Greece; Staedtisches Klinikum Karlsruhe, Karlsruhe, Germany; University Hospital Grenoble Alpes and Grenoble Alpes University, Grenoble, France; Catharina Ziekenhuis, Eindhoven, The Netherlands; Department of Cardiology, Medical University Hospital Heidelberg, Heidelberg, Germany; Department of Cardiology, Medical University of Warsaw, Warsaw, Poland; České Budějovice Hospital and Faculty of Health and Social Sciences, University of South Bohemia in České Budějovice, České Budějovice, Czechia; RWTH University Hospital Aachen, Aachen, Germany; Department of Cardiology—Electrophysiology, University Hospital of Münster, Münster, Germany; Institut Jantung Negara, Kuala Lumpur, Malaysia; Cardiology Department, Royal Papworth Hospital and Department of Medicine, University of Cambridge, Cambridge, UK; Department of Cardiology, Sint-Jan Hospital, Bruges, Belgium; Hospital Universitario y Politécnico La Fe, Valencia, Spain; Boston Scientific, Marlborough, MA, USA; Boston Scientific, Marlborough, MA, USA; Boston Scientific, Marlborough, MA, USA; Boston Scientific, Marlborough, MA, USA; Cardiology Department, St. Antonius Hospital, Nieuwegein, The Netherlands; Amsterdam University Medical Center, Amsterdam, The Netherlands

**Keywords:** Pulsed Field Ablation, Atrial Fibrillation, Real-World, First-Line

## Introduction

Atrial fibrillation (AF) is the most commonly diagnosed sustained arrhythmia worldwide, estimated to affect over 50 million patients.^1^ Catheter ablation has emerged as a safe and effective treatment strategy for the management of AF, with pulsed field ablation (PFA) rapidly growing in adoption due to its excellent safety profile.^2,3^ Historically, antiarrhythmic drugs (AADs) have been prescribed as an initial rhythm control strategy.^4^ However, after success in clinical studies, new guidelines for AF suggest catheter ablation as a first-line rhythm control therapy in paroxysmal AF (PAF) patients.^5–8^

The global FARADISE registry was designed to evaluate safety and efficacy outcomes in the context of a real-world, standard-of-care registry. This sub-analysis evaluates outcomes in AF patients receiving PFA as first-line ablation compared with those receiving PFA after AAD treatment.

## Methods

FARADISE is a prospective, non-randomized, single-arm registry enrolling subjects clinically indicated for an AF ablation procedure using the pentaspline PFA catheter.^9^ Eligible subjects included those who were clinically indicated for AF ablation with the FARAPULSE PFA system per the physician’s medical judgement and per the hospital’s standard of care. The complete methods have been described previously.^9^

### Procedure

Procedures were performed using the pentaspline PFA catheter (Farawave™, Boston Scientific, Marlborough, MA) and a steerable sheath (Faradrive™, Boston Scientific, Marlborough, MA). PFA applications were delivered with a PFA generator (Farastar™, Boston Scientific, Marlborough, MA) with a voltage output of 1.8–2.0 kV.

### Analysis

Patients were stratified as those undergoing PFA after a history of Class I/III AAD treatment and those receiving PFA as first-line therapy. Patients receiving a repeat ablation were excluded from this analysis. Clinical effectiveness was defined as freedom from acute procedural failure, documented atrial fibrillation/atrial flutter/atrial tachycardia (AF/AFL/AT) recurrence, repeat procedure, and cardioversion. The endpoint was assessed after a 90-day blanking period post index procedure.^9^ The clinical effectiveness definition allowed for AAD use during the 1-year follow-up.

### Follow-up

Full endpoint definitions have been described previously.^9^ Patients have follow-up visits at pre-discharge, 3, 6, 12, 24, and 36 months. Arrhythmia recurrence will be monitored using each hospital’s standard of care with the recommendation that patients receive 24-h Holter monitoring at 3 months and again at 1-, 2-, and 3-year follow-up. One-year outcomes are reported here.

### Statistics

The complete statistical methods have been described previously.^9^

## Results

### Patient demographics

In total, 1158 AF patients were treated with the pentaspline PFA catheter, 1043 of which were *de novo* ablations. Of those, 548 received PFA as first-line treatment (358 [65.3%] PAF; 176 [32.1%] female; 63 ± 11 years) and 495 patients received PFA after Class I/III AAD use (336 [67.9%] PAF; 172 [34.7%] female; 64 ± 10 years) (*Figure 1A*). Baseline and procedural characteristics were similar between groups.

**Figure 1:**
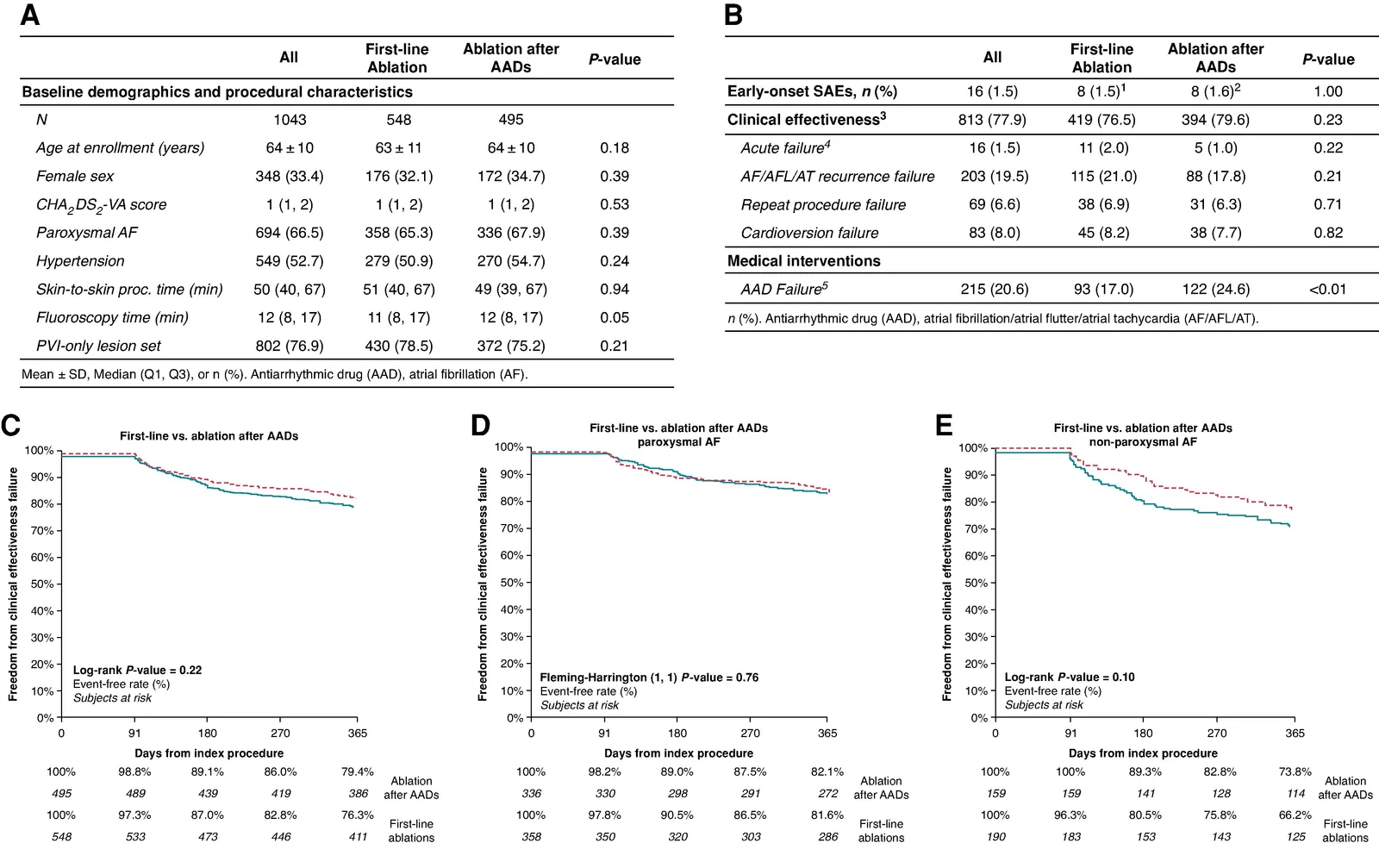
(*A*) Baseline demographics and procedural characteristics in first-line patients and patients receiving PFA after prior Class I/III AAD use. (*B*) Serious adverse events (SAEs) and clinical effectiveness components in first-line patients and patients receiving PFA after prior Class I/III AAD use. Kaplan-Meier estimates of one-year clinical effectiveness comparing first-line PFA to PFA after AAD history in (*C*) all *de novo* patients, (*D*) *de novo* paroxysmal AF patients, and (*E*) *de novo* non-paroxysmal AF patients. **^1^**TIA (1 event), cardiac tamponade/perforation (1), pericarditis (1), vascular access complication (5). **^2^**Cardiac tamponade/perforation (3 events), pericarditis (1), pulmonary oedema (1), vascular access complication (3). **^3^**Subjects can have more than one effectiveness failure component. **^4^**There were 16 patients who met the criteria for acute failure due to entrance block testing not being performed in one or more treated PVs. **^5^**Includes amiodarone.

### Safety events

The one-year safety outcomes from the complete FARADISE cohort have been previously published.^9^ In this sub-analysis consisting of a *de novo* cohort, the number of serious adverse events was low in both cohorts, including 8 (1.5%) in first-line patients and 8 (1.6%) in AAD history patients (*P* = 1.0) (*Figure 1B*). No death, myocardial infarction, persistent phrenic nerve injury, or stroke occurred through 12 months.

### Clinical effectiveness

Clinical effectiveness was favourable in both first-line patients (76.3%) and patients undergoing PFA after AAD history (79.4%, *P* = 0.22) (*Figure 1C*). There was no difference when further divided by AF indication (PAF: first-line 81.6% vs. AAD history 82.1%, *P* = 0.76; non-PAF: first-line 66.2% vs. AAD history 73.8%, *P* = 0.10, *Figure 1D* and *E*).

Within the full *de novo* patient cohort, first-line PFA patients had fewer new or escalating AAD events (including amiodarone) during follow-up than PFA patients with prior AAD use (93 [17.0%] vs. 122 [24.6%], *P* < 0.01). The risk of AF/AFL/AT recurrence (115 [21.0%] vs. 88 [17.8%], *P* = 0.21), repeat procedures (38 [6.9%] vs. 31 [6.3%], *P* = 0.71), and cardioversions (45 [8.2%] vs. 38 [7.7%], *P* = 0.82) were similar in patients receiving PFA as first-line therapy compared to those receiving PFA after prior AAD use (*Figure 1B*).

## Discussion

### Main findings

The FARADISE registry successfully enrolled and treated over 1000 patients in 21 countries, capturing real-world outcomes from Europe, the Middle East, and Asia Pacific. Safety events were rare, and patients experienced robust clinical effectiveness, regardless of AAD history or AF indication. Notably, baseline characteristics were comparable between groups, suggesting similar patient profiles.

### Efficacy of first-line therapy

Among patients with symptomatic PAF, first-line catheter ablation is associated with superior rhythm control as well as a reduction in long-term recurrence compared to AADs and is now the recommended treatment modality in certain patient populations.^6–8,10^ While AADs are the standard initial treatment strategy for AF, AADs have narrow therapeutic windows, and many patients report negative side effects.^4^ Despite this evidence, AADs may be preferred for patients unable to undergo an ablation procedure or for patients with limited access to electrophysiology clinics.

### AAD failures

Analysis of the FARADISE registry highlights the important distinctions in AAD failures between groups. First-line patients were less likely to experience AAD failures, including amiodarone. Regional variation in clinical practice may play a role in AAD and amiodarone failures. Further, physicians may prescribe amiodarone earlier in patients who have already failed another AAD, aligning with contemporary guideline recommendations.^5^ These findings emphasize the relationship between treatment history and disease severity in relation to treatment strategy in AF.

### Limitations

This analysis has intrinsic limitations due to selection bias in patients undergoing PFA after prior AAD use, as patients who responded to AAD treatment may not be referred for an ablation. These patient groups are not randomized and thus could have differences in AF duration, substrate severity, and referral patterns. This analysis allows for AAD use during follow-up, which may limit direct comparisons to other PFA studies. Further, this registry relies on 24-hour Holter monitoring, which may underestimate AF recurrence compared with implantable loop recorders. Lastly, as a standard-of-care registry, treatment approaches may vary by physician and geographic location, reflecting individual preferences and institutional protocols.

## Conclusions

The current study was associated with comparable safety and clinical effectiveness, both for first-line AF patients and AF patients undergoing PFA after prior AAD use. These outcomes were consistent among PAF and non-PAF patient subpopulations, highlighting the flexibility in treatment for AF patients.

## Data Availability

The data and study protocol for this clinical study may be made available to other researchers in accordance with Boston Scientific’s data sharing policy (https://www.bostonscientific.com/en-US/data-sharing-requests.html).
